# Brain Tissue Damage Induced by Multimodal Neuromonitoring In Situ during MRI after Severe Traumatic Brain Injury: Incidence and Clinical Relevance

**DOI:** 10.3390/jcm11113169

**Published:** 2022-06-02

**Authors:** Daniel Pinggera, Paul Rhomberg, Ronny Beer, Claudius Thomé, Ondra Petr

**Affiliations:** 1 Department of Neurosurgery, Medical University Innsbruck, 6020 Innsbruck, Austria; claudius.thome@tirol-kliniken.at (C.T.); ondra.petr@yahoo.com (O.P.); 2 Department of Neuroradiology, Medical University Innsbruck, 6020 Innsbruck, Austria; paul.rhomberg@tirol-kliniken.at; 3 Department of Neurology, Medical University Innsbruck, 6020 Innsbruck, Austria; ronny.beer@tirol-kliniken.at

**Keywords:** magnetic resonance imaging, severe traumatic brain injury, neuromonitoring

## Abstract

Both neuromonitoring and early magnetic resonance imaging (MRI) provide crucial information for treatment management and prognosis in patients with severe traumatic brain injury (sTBI). So far, neuromonitoring in situ impedes the routine implementation of MRI due to safety concerns. We aimed to evaluate the brain tissue damage induced by inserted neuromonitoring devices and its clinical relevance. Nineteen patients with sTBI and being exposed to at least one MRI with neuromonitoring in situ and one follow-up MRI after neuromonitoring removal were analyzed. All MRIs were reviewed for specific tissue damage. Three females and sixteen males (aged 20–74 years, mean 42.8 years) with an initial median GCS of 5 (range 3–8) were analyzed. No lesion was observed in six patients (31.6%), whereas another six patients (31.6%) demonstrated a detectable probe trajectory. Probe-related tissue damage was visible in seven patients (36.8%) with the size of the lesion prone to further enlarge with increasing cumulative duration of MRI examinations. Upon interdisciplinary evaluation, the lesions were not considered clinically relevant. Neuromonitoring probes in situ during MRI examinations may cause local brain tissue damage, yet without any clinical implications if placed correctly. Therefore, indications must be strictly based on joint decision from all involved disciplines.

## 1. Introduction

Continuous cerebral multimodal neuromonitoring has proved to improve both outcome and treatment in critically ill neurosurgical patients, especially after severe traumatic brain injury (sTBI) and subarachnoid hemorrhage (SAH) [1,2,3,4,5,6]. Another integral element in the diagnostic and therapeutic management of these patients is advanced neuroimaging. Early magnetic resonance imaging (MRI) is salutary, providing in-depth analysis of traumatic intracranial lesions with not only structural assessment of prognosis, but also pertinent information for further treatment management [7,8,9,10].

Staying oftentimes on prolonged ventilation, cerebral neuromonitoring is required in the first critical days up to 2–3 weeks in patients with sTBI. Since robust clinical data on MR-safety for most neuromonitoring probes at 1.5 and 3 Tesla conditions are still lacking, many clinicians and radiologists are reluctant to perform the MRI at this time point, thus losing a germane diagnostic tool. The terms MR conditional and MR (un)safe were introduced by the American Society for Testing Materials International (ASTM) to help clarify how an object may be used safely in the MR environment. An MR-safe item poses no known hazards in all MR imaging environments. An MR-conditional device poses no hazard if it is used in a specified MR environment with specified conditions of use. If an item is designated MR unsafe, it is said to pose a hazard in all MR imaging environments. A major potential risk of neuromonitoring probes in situ during the MRI examination is a heating of the metal tip, resulting in potential thermal injuries [11,12,13,14,15,16,17]. Hence, neuromonitoring interferes in indications for early MRI in daily clinical practice.

On the other hand, removal with new replacement of neuromonitoring afterwards due to a scheduled early MRI seems not to be justified, since continuous neuromonitoring is in the meantime interrupted, and subsequently the sedation levels may have to be increased for the replacement. Additionally, neuromonitoring-related hemorrhage occurs in 2 to 8%, with increasing risk in coagulation abnormalities, impeding the indications for this alternative [18,19].

As sequalae to a prospective imaging study on cerebral metabolism using advanced MR imaging methods, in some patients, we detected brain tissue lesions in retrospect, potentially inflicted by neuromonitoring [20,21,22]. Therefore, to estimate the risk of MRI with neuromonitoring probes in situ, we aimed to evaluate the specific brain tissue damage induced by neuromonitoring devices in situ and its clinical relevance.

## 2. Materials and Methods

### 2.1. Patients

Patients aged 18 to 85 years with sTBI and an initial Glasgow Coma Scale (GCS) score of 8 or less who required treatment according to the Brain Trauma Foundation guidelines, including insertion of multimodal neuromonitoring, were included in this retrospective analysis from another prospective imaging study described elsewhere in detail. In brief, an advanced MRI protocol including phosphorous magnetic resonance spectroscopy (^31^P-MRS) was performed at two different time points to reveal alterations in cerebral energy metabolism [20,21,22]. All patients underwent at least one early MRI within the first 14 days after the initial sTBI with the neuromonitoring in situ, followed by at least one MRI examination after removal of the intracranial neuromonitoring probes.

Exclusion criteria were contraindications to MRI and/or an unstable clinical condition disqualifying the patient from undergoing an MRI examination safely. The study protocol was approved by the local ethics committee of the Medical University Innsbruck (Protocol number: AN 1443/2021). Written informed consent was obtained from all included patients and/or from their legal guardians as part of the initial study (AN2014-0201 339/4.6).

Nineteen patients with severe TBI (mean GCS 5, range 3–8) admitted to our Department from March 2015 to December 2017 met the inclusion criteria and were analyzed.

Patient outcome was assessed systematically at routine follow-up visits 6/12 months after discharge or via medical reports/online records from the neurorehabilitative departments. The final Glasgow Outcome Score (GOS) score available for each patient between 6 and 12 months after discharge, was used for further analysis.

### 2.2. Intracranial Probes

ICP was measured using a Neurovent-P probe (Raumedic AG + CO, Raumedic, Germany). Brain tissue oxygen tension (PbtO2) was monitored using a Licox^®^ CC1.SB probe (Integra^®^ LifeSciences Corp, Plainsboro, NJ, USA). The ICP probe is rated MRI conditional, both on 1.5 and 3.0 Tesla, whereas the Licox^®^ CC1.SB probe has not been tested for compatibility with MR systems, in contrast to the longer the Licox^®^ CC1.P1 probe (Integra^®^ LifeSciences Corp, Plainsboro, NJ, USA), which is rated MR conditional on 1.5T.

External ventricular drainage was inserted if indicated, using a Raumedic ventricular catheter (Raumedic AG + CO, Raumedic, Germany), and it was considered MRI safe as being manufactured out of plastic.

All probes were inserted under sterile conditions either in the operating room or bedside in the neurosurgical intensive care unit. During MRI examinations, the ICP probe was placed in a coil-like configuration according to the manufacturer’s specifications.

### 2.3. Magnetic Resonance Imaging

All patients underwent MRI including the following sequences: 3D T1 MPRAGE, 3D T2-space, FLAIR, DWI, 3D SWI, pASL, and 3D TOF. MRI was performed on a 3 Tesla whole-body system (Verio, Siemens Medical AG, Erlangen, Germany). As part of another analysis, ^31^P-MRS was performed on the same MRI unit with a double-tuned 1H/31P volume head coil (Rapid Biomedical, Würzburg, Germany) in all patients [20]. Of note, mean duration of the MRI scan, including positioning the patient on the MRI table and back plus installing the monitoring, amounted to 82 min (range 45–120 min) [20]. MRIs were scheduled within 14 days after initial traumatic event and then 6 months thereafter. Further MRIs were performed as indicated.

Substantial lesions in the follow-up MRIs were defined as substance defects in 3D T1 MPRAGE, FLAIR and/or 3D T2-space bigger than the size of the probe tip (i.e., for ICP: 1.6 × 1.6 × 3 mm, for PbtO2: 1 × 1 × 2 mm). Signals of probe insertions solely, e.g., the trajectory of an inserted probe, with no defect more than the latter probe tip size, were surveyed, yet defined as no thermal injury.

Each MRI was reviewed in detail by both board-certified neuroradiologists and three neurosurgeons. The latter also analyzed MRI for the clinical relevance of the brain damage induced using a dichotomous form (Yes/No).

### 2.4. Statistical Analysis

Statistical analysis was conducted using IBM SPSS Statistics (V.21, version 21, SPSS Inc., IBM, Chicago, IL, USA). The distribution of categorical variables was assessed with a chi-square and a Fisher’s exact test. Differences with a *p*-value of less than 0.05 were considered statistically significant.

## 3. Results

### 3.1. Patients

A total of 19 patients (aged 20–74 years, mean 42.8 years; 3 female and 16 male patients) with sTBI were reanalyzed. All patients underwent at least one early MRI with intracranial neuromonitoring probes, along with at least one follow-up MRI after probe removal. The indication of the initial MRI was based on an interdisciplinary discussion of the needs and benefits of the diagnostic procedure.

Median GCS on admission was 6 (range 3–8). For insertion of neuromonitoring, a single burr-hole was performed in nine patients (47.4%), osteoclastic craniectomy was used in six patients (31.6%), and osteoplastic craniotomy in four patients (21.1%). Further detailed demographic data are summarized in Table 1.

Mean GOS at 6 months was 4 (range 1–5) and mean extended GOS at 6 months was 6 (range 1–8).

### 3.2. Intracranial Probes

ICP probes were inserted in all 19 patients with additional placement of PbtO2 probes in 13 cases (68.4%). All probes were positioned parallel to one another in the frontal white matter via one burr hole. The probes were tunneled in 16 cases (84.2%) according to our in-house protocol [23]. A bolt system (RAUMEDIC Bolt Kit CH9) was used in three cases (15.8%). External ventricular drainage for hydrocephalus was required only in one patient (5.3%) (Table 1).

Whilst the ICP course after MRI examination showed no relevant alterations, no technical or other neuromonitoring issues occurred and probe replacement was not necessary.

### 3.3. Imaging

After insertion of neuromonitoring, all patients routinely underwent an early cerebral CT scan in order to verify the correct probe position and to rule out probe-related hemorrhage. No malposition or neuromonitoring-related hemorrhages were noticed in our study population.

Twelve patients (63.2%) underwent a single early MRI with the probes in situ, two MRIs with inserted neuromonitoring were performed in six patients (31.6%), and one patient (5.3%) received three MRI examinations with implanted probes. All 19 patients underwent one follow-up MRI after probe removal. Median time to follow-up MRI was 181 days (range 11—335 days, ±103 days).

**Figure 1:**
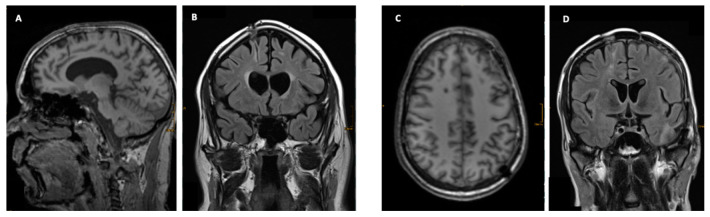
Examples of two patients without a lesion, but with a visible trajectory after probe removal. Panel (**A**,**C**) present T1-weighted MRI with corresponding coronal imaging (Panel (**B**,**D**)).

**Figure 2:**
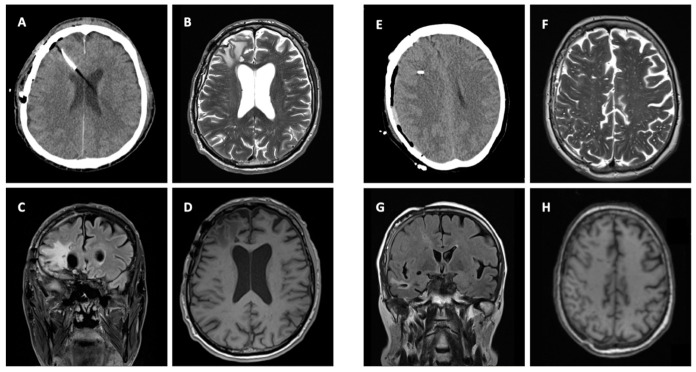
Two exemplary MRI lesions after removal of ICP probe. Panel (**A**,**E**) show a cerebral CT scan with probes in place. Panel (**B**,**F**) show axial T2-weighted MRI after removal of the probe. Panel (**C**,**D**,**G**,**H**) show corresponding Flair MRI after probe removal.

**Figure 3:**
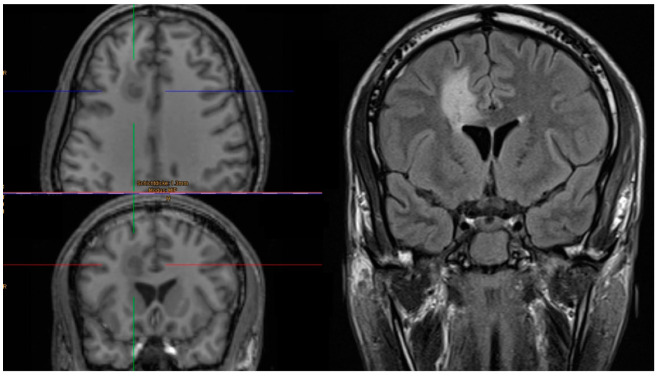
Axial T1-weighted MRI with corresponding coronal imaging showing a lesion in right frontal lobe. Coronal FLAIR demonstrating local edema. The patient underwent 3 MRI examinations with an ICP probe in situ.

**Figure 4:**
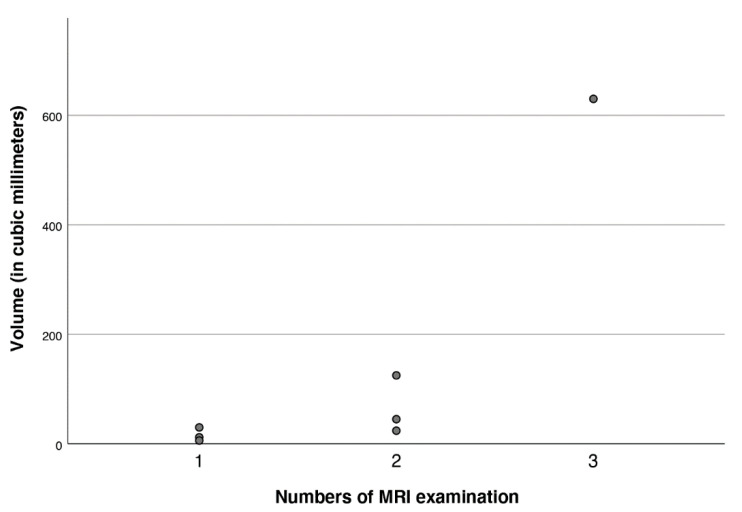
Volumes (in cubic millimeters) of the brain tissue lesions in relation to the numbers of MRI examinations.

Neuromonitoring-induced spheric brain tissue damage was visible in seven patients (36.8%), signs of probe trajectory only were detected in six patients (31.6%), and no lesion was observed in six patients (31.6%). All identified lesions were visible in both 3D T1 MPRAGE and FLAIR with the largest diameter of 10 mm located at the probe tip (Table 1 and Figure 1). Of note, all noticeable lesions were detected on the location of the tip of the ICP probes, verified in an early cerebral CT scan, whereas no lesion was related to the inserted PbtO2 probes. Probe trajectories were detectable in both 3D T1 MPRAGE and FLAIR but with no additional brain damage around the probe tip (Figure 1 and Figure 2). The largest lesion (10 × 9 × 7 mm) was observed in a male patient in whom three early MRI examinations were performed (Figure 3 and Figure 4). Taking heed of counts of performed MRI examinations with neuromonitoring probes in situ, patients receiving two MRI examinations (*n* = 6) showed specific brain tissue damage in 50% (*n* = 3), compared to patients undergoing only one MRI where the related brain tissue damage was apparent in 25% (*n* = 3). Further details are presented in Table 1.

### 3.4. Clinical Signicance

Each MRI was reviewed in detail by three board-certified neurosurgeons (DP, OP and CT). All lesions (both spheric brain tissue damage and probe trajectory, *n* = 13) were classified as clinically irrelevant by all three raters (100% not clinically relevant). This was justified by the given size of the lesions and considering the overall brain damage owing to initial sTBI (Figure 4). Additionally, the location of the probes, respective to the tissue damage, was unexceptional in the frontal white matter, presenting a non-eloquent location (Table 1).

There were no statistically significant differences in neurological outcome (GOS, favorable vs. unfavorable outcome) between the patients with the brain tissue damage and without (*p* = 0.259).

## 4. Discussion

Our study on sTBI patients who received an early MRI with neuromonitoring probes in situ provides comprehensive data on related radiographic and clinical sequelae. Especially for the ICP probes under a 3 Tesla environment, a specific thermal brain tissue damage located along the ICP probe tip occurred in approximately one third of our study population. Interestingly, albeit not tested for MR safety, the PbtO2 probes implanted in our series appeared to generate no visible brain lesions in any of the included patients.

We also discerned an evident relationship between the time spent in the MRI and the size of the induced lesion; in particular, the longer the time of the examination the larger the size of the damage. However, apart from radiographic manifestations, no further harm occurred with any other substantive impact. To our best knowledge, this is the first study demonstrating specific brain damage induced by neuromonitoring probes in situ during MRI examinations in a well-defined patient population and its clinical sequelae.

Both continuous ICP and PbtO2 monitoring are central in neurocritical care. ICP management has become a cornerstone in sTBI care [3,4,24,25]. Additionally, maintenance of adequate brain oxygenation is one of the most important goals in neurocritical care, and the assessment of current tissue oxygenation is essential for patient management as hypoxia can aggravate secondary brain damage. The clinical benefit of brain tissue oxygenation monitoring has been well documented in TBI and SAH patients [26,27,28,29,30]. Importantly, neuromonitoring devices provide safe and accurate monitoring within 7 to 10 days without increased monitoring-related morbidity [31]. Summarizing, neuromonitoring is considered to be crucial, but can complicate MRI examinations due to safety concerns. This can be a very unpleasant constellation, as MRI repeatedly provides pivotal information in patients with acute brain injury. MRI scans are often used to facilitate the clinical management and further decision making by, e.g., assessing perfusion deficits or other aspects of TBI [20,32,33,34,35,36].

Thermal damage of brain tissue during MRI procedures caused by ICP probes are rarely written about. Tanaka et al., reported a case series of seven patients, in which thermal brain injuries with ICP transducers (Codman MicroSensor) occurred. In their conclusion they stressed the importance of strictly adhering to safety guidelines [17]. Their presented case series also showed a high amount of probe malfunctioning after MRI, which was not seen in any of our cases [22]. In an experimental study by Coles et al., another probe (i.e., Ventrix parenchymal intracranial pressure monitoring probe, Integra Neurosciences^®^, Plainsboro, NJ, USA) modified using a fiber-optic extension cable was tested on 3.0 Tesla MRI. No rise in temperature was observed [37]. A significant proportion of the damage may be caused by repeated or long-lasting MR examinations. In our study we could correlate the proportion of the damage to repeated or long-lasting MR examinations. The largest lesion around the probe tip was recorded in a patient undergoing three MRIs with ICP monitoring in situ. In our search for the cause, we noticed that our experimental MR protocol exceeded the safety guidelines for examination time set by the manufacturer. Other explanations could be the use of ^31^P-MRS, which is not mentioned in the guidelines, but has probably never been tested for.

Data on brain damage induced by PbtO2 monitoring are rare, reporting a temperature increase beyond 4 °C under a 3 Tesla environment, posing a significant risk for tissue brain damage using the MR conditional Licox^®^ probe CC1.P1 [38]. Our study did not show any radio-frequency-induced thermal damage due to the shorter PbtO2 probe (Licox^®^ CC1.SB probe). This is also in line with data of Ercole showing safety concerns about the use of Licox^®^ CC1.P1 probes in a 3T environment. However, in the same article he reports that the CC1.SB probe, which has a different resonant frequency, has been safely used for some years in his department in a 3T MR setting [39].

Tissue brain damage was expected to be more likely in our cohort, considering a possible enhancing effect of the increased heating due to a proximity of ICP and PtbO2 probes. However, we could not detect any radio-frequency-induced thermal damage along the PbtO2 probe’s location, but we cannot rule out a negative influence on the electromagnetic properties of the ICP probe.

Our incidence of MR-detectable visible lesions of 36.8% is high, albeit clinically irrelevant. On the other hand, the occurrence of these lesions may be generally underestimated, solely for the lack of searching for it in routine follow-up MRIs after sTBI. Additionally, lesions caused by neuromonitoring may be misinterpreted as a sequel of initial brain injury. Importantly, our patient population was part of a prospective MRI study with very extensive imaging, which surpasses common MRI duration, and thus potentially exacerbating injury [20,21]. Although officially rated as MR-conditional, some neuromonitoring devices can cause harm when used in an experimental design. This issue must be addressed in patient information and ethics committee sessions.

MRI safety issues arise also in other fields of neurosurgery. In deep-brain stimulation, there were two reported cases with thermal brain injury, both caused by failure to strictly follow safety recommendations [15]. However, when abiding by correct specifications, MRI in DBS is considered safe [13,14,15,16]. In some cases of epilepsy, intra-cranial EEG electrodes require subsequent MRI for post-implantation localization. It was demonstrated that it can pose a significant risk for radio-frequency-induced thermal injury, however only in a small number of patients [40,41].

We acknowledge that our study has several limitations. Certainly, our results must be interpreted carefully considering the small sample size, even though it is one of the largest in the pertinent literature. Additionally, our initial analysis focused primarily on cerebral energy metabolism using ^31^P-MRS, but not on brain tissue damage [20]. As stated above, advanced MRI including MR spectroscopy was performed in our patient cohort, very likely overestimating thermal brain damage due to neuromonitoring in situ. Retrospectively, neuromonitoring probes used in our study were not specifically tested under our extensive applied advanced neuroimaging protocol.

As a very first consequence after our first interim analysis, we tried to enhance patients’ safety by establishing a standard operating procedure for MRI with neuromonitoring probes including the following steps:

1. Stringent indications for all MRI examinations in neurointensive care patients in a close coordination between neuroradiologists and neurointensivists/neurosurgeons.
2. Early cerebral CT scan to determine the accurate location of neuromonitoring probes, detecting malposition (i.e., eloquent areas), and/or neuromonitoring-related surgical complications.
3. MRI protocol adjustments (i.e., reduced number of applied MR sequences depending on underlying questions, e.g., only DWI, 3D SWI, T2, T1; reduction in specific absorption rate; greater slice thickness, etc.).
4. Pre-imaging removal or reduction of neuromonitoring probes to avoid all potential enhancing effects due to the proximity of multiple probes. Currently, we perform MRI only with one ICP probe in situ.
5. No use of bolt systems in patients with severe TBI to avoid artefacts and problems with patient positioning in the head coil.

## 5. Conclusions

Neuromonitoring probes in situ may cause a local thermal brain tissue damage during an MRI examination in a considerable number of patients. Yet, we could not verify any clinical implications for the patient from the resulting brain tissue damage due to the location of the lesion and the sequelae of the sTBI.

Neurointensivists should be aware of this issue; however, neuromonitoring in situ should not disqualify per se patients from standard MRI examinations in the early phase after sTBI if needed.

Finally, both further communication on the individual standards of MR safety and real-world use are needed and further clinical trials with larger patient populations are warranted.

## Figures and Tables

**Table 1:** Demographics of study cohort including age, gender, kind of probes implanted, kind of surgery, kind of lesion, numbers of MRIs performed, side of probe placement, size of visible lesion in millimeter, and Glasgow Outcome Score.

Patient-#	Sex	Age at Trauma	ICP	PbtO2	EVD	Surgery	Lesion	Number of MRIs with Probes	Probes Localization	Size of Lesion (Length × with × Height, in mm)	GOS
1	male	35	yes	yes	no	Open/tunneled	No Lesion	1	Frontal, left		4
2	male	27	yes	no	no	Bolt	Lesion	3	Frontal, right	9 × 10 × 7	4
3	male	49	yes	yes	no	Open/tunneled	Lesion	2	Frontal, left	5 × 3 × 3	5
4	female	47	yes	yes	no	Open/tunneled	No Lesion	1	Frontal, right		5
5	male	60	yes	yes	no	Open/tunneled	Probe trajectory	1	Frontal, right		5
6	male	20	yes	no	no	Bolt	Lesion	1	Frontal, right	5 × 3 × 2	5
7	male	41	yes	yes	no	Open/tunneled	Probe trajectory	1	Frontal, right		4
8	male	56	yes	yes	no	Open/tunneled	No Lesion	1	Frontal, left		5
9	male	55	yes	yes	no	Bolt	Lesion	1	Frontal, right	3 × 2 × 2	1
10	male	74	yes	yes	no	Open/tunneled	Lesion	2	Frontal, right	5 × 5 × 5	5
11	female	49	yes	no	no	Open/tunneled	No Lesion	2	Frontal, right		4
12	male	32	yes	no	no	Open/tunneled	No Lesion	1	Frontal, left		5
13	male	46	yes	yes	no	Open/tunneled	No Lesion	2	Frontal, right		3
14	male	34	yes	yes	no	Open/tunneled	Lesion	2	Frontal, right	4 × 3 × 2	4
15	male	29	yes	yes	yes	Open/tunneled	Probe trajectory	1	Frontal, right		5
16	male	21	yes	yes	no	Open/tunneled	Probe trajectory	1	Frontal, right		5
17	female	69	yes	yes	no	Open/tunneled	Probe trajectory	2	Frontal, right		4
18	male	30	yes	no	no	Open/tunneled	Lesion	1	Frontal, left	3 × 2 × 1	4
19	male	40	yes	no	no	Open/tunneled	Probe trajectory	1	Frontal, right		3

## Data Availability

Not applicable.
